# A comprehensive and accessible database of jawed-vertebrate ohnologs

**DOI:** 10.1126/sciadv.adz8270

**Published:** 2026-08-07

**Authors:** Łukasz Niezabitowski, Róisín Long, Anthony K. Redmond, Aoife McLysaght

**Affiliations:** ^1^ Smurfit Institute of Genetics, Trinity College Dublin, Dublin 2, Ireland.; ^2^ School of Medicine, University College Dublin, Dublin 4, Ireland.

## Abstract

Whole-genome duplications (WGDs) profoundly shaped early vertebrate evolution. The set of paralogs retained from these events—“ohnologs”—has had lasting impacts on genome structure and function, including disease gene enrichment. Despite this, we still lack a gold standard ohnolog database. Here, we have harnessed ancestral genome reconstructions together with phylogenetic and synteny information to produce a robust ohnolog dataset that includes details on the nature and quality of evidence and a user-friendly interface. We have been able to resolve the 1R versus 2R origins of a large number of cases, as well as including previously hard-to-detect ohnologs. We find that 1R-retained ohnologs are involved in cellular transport, while those retained after both 1R and 2R are biased toward signaling functions, especially neuronal signaling. These findings suggest that after an initial adaptation to the cellular burden of polyploidy, WGD expanded the opportunities for intercellular communication.

## Teaser

A reliable ohnolog database yields insights into vertebrate evolution.

## INTRODUCTION

Whole-genome duplications (WGDs) are major evolutionary events whereby all genomic contents are doubled (*1*). This not only creates large amounts of raw material for evolution but also preserves any regulatory networks and gene expression ratios, thus creating unique evolutionary opportunities (*2*–*5*). Paralogs retained after WGD are known as “ohnologs” (*1*, *6*), and these constitute a nonrandom and particularly interesting subset of the genome, being enriched for developmental genes, transcription factors, protein complex membership, dosage-sensitive genes, and genes involved in human disease (*7*–*11*).

WGD and ohnologs are of interest to a broad range of fields. In terms of molecular evolution, WGD is often credited with creating the circumstances that facilitated massive species radiations. It has been observed that the duplication of developmental genes, most notably the *Hox* genes, is almost exclusively achieved through WGD (*7*, *12*, *13*). Novel genes created by WGD have recently been shown to be prone to lineage-specific resolution (*14*–*16*), which may be a previously unappreciated factor in lineage-specific adaptations. Ohnologs that have been retained over long evolutionary periods are enriched for dosage-sensitive genes, a characteristic that makes them more likely to be involved in human disease when disrupted (*8*, *9*, *17*). This has the result that knowing the evolutionary history of genes of interest in disease can be helpful in both narrowing down the candidate gene list and in understanding the pathobiology.

While genome duplications are best characterized in plants (*18*–*20*), there are multiple examples in vertebrates (*6*, *8*, *16*, *21*–*24*), most famously the 2 Rounds (2R) that occurred at the base of the lineage. We now know that the first event (dubbed 1R) is shared by all vertebrate genomes and was shortly followed by 2R in jawed vertebrates (gnathostomes) with cyclostomes experiencing an independent event (*4*, *5*, *25*, *26*). These genome duplications have been implicated in major vertebrate innovations, including the development of the adaptive immune system (AIS) and an increase in morphological diversity (*4*, *27*–*30*). As such, there is a huge interest in identifying and analyzing ohnologs across vertebrate genomes to expand our understanding of vertebrate biology.

Following a WGD, the initial gene order and content on homeologous chromosomes will be nearly identical (*1*). However, with evolutionary time, this pattern becomes degraded by chromosome fusion, fission, translocation, and loss events, as well as internal chromosomal rearrangements and gene loss events (*2*, *14*–*16*, *25*, *26*, *31*). Despite this, we can still uncover the traces of the formerly symmetrically duplicated genome in the form of collinear blocks of paralogs (*10*, *11*, *21*, *22*, *25*, *26*, *32*, *33*). These long stretches of conserved gene order and content, termed microsynteny blocks, are a distinctive feature of WGD and have been leveraged in previous work on vertebrate ohnolog identification. Similar patterns are also apparent on a macroscale, i.e., over longer chromosomal segments and ignoring gene order (*10*, *26*).

Despite this, generating comprehensive ohnolog datasets has proved challenging. Although synteny is a highly reliable marker, it becomes degraded over time. This is especially relevant for ancient duplications such as 1R and 2R (*26*, *31*, *34*, *35*). Alternative approaches may rely on phylogenetics to infer the duplication timing of putative ohnologs (*4*, *5*, *36*). However, early work found tree topologies that were highly inconsistent, perhaps due to poor taxon sampling and stochastic errors, leading some to reject the “2R hypothesis” (*37*) and others to question the number and/or timing of WGD events (*38*, *39*). Further complicating matters, the tendency of cyclostome genes to cluster outside of gnathostome clades because of compositional heterogeneity makes them unreliable as an outgroup to 2R (*4*, *5*, *40*–*42*). The possibility of delayed and asynchronous rediploidization after WGD contributes additional complexity, as different gene families may have different, although correct, tree topologies even though they expanded as part of the same WGD event (*15*, *16*). Phylogenetic and synteny-based approaches are also complicated and time-consuming, leading to many vertebrate ohnolog datasets to be created ad hoc and relying primarily on sequence similarity (*8*, *22*, *36*). This has resulted in a high number of mutually inconsistent datasets that often lack adequate documentation, hampering their use for subsequent analyses, and with only minor success in distinguishing between 1R and 2R duplicates, none on a genome-wide scale.

**Fig. 1.**
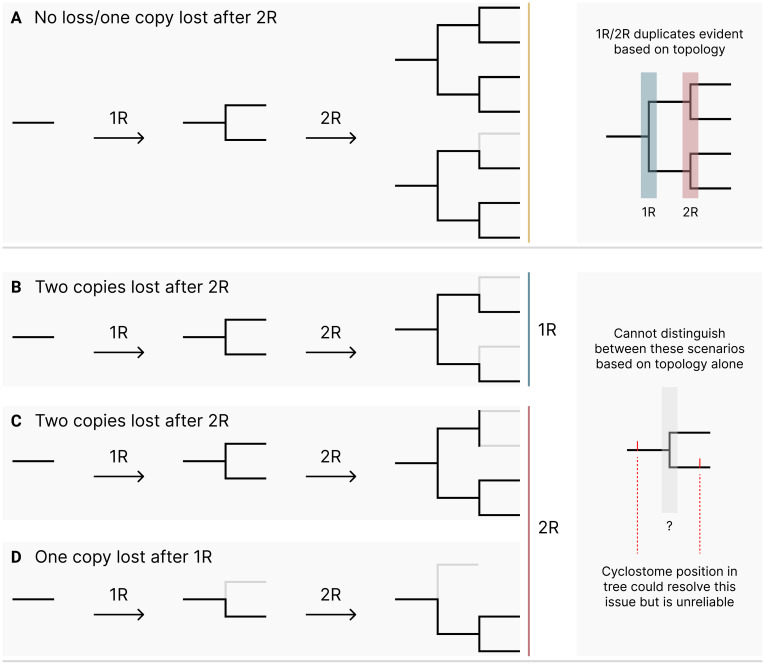
Phylogenetic-based approaches are insufficient to disentangle 1R and 2R duplicate pairs. Various WGD (bifurcation) and gene loss (grayed-out branch) scenarios are shown on the left, and the resultant gene tree topologies are shown on the right. Because 1R and 2R are defined by relative timing, any gene family tree that includes at least two WGD nodes allows the identification of 1R (earlier) and 2R (later) relationships. This is only the case for families with at least three of the four ohnologs retained (**A**). When only two of the four ohnologs are retained (**B** to **D**), the tree topologies are identical, even though the retained pair may have duplicated in 1R (B) or 2R (C and D). The inclusion of a cyclostome outgroup could theoretically solve this problem as this lineage diverged in the interval between 1R and 2R. A cyclostome branch before the gnathostome duplication would indicate that the retained pair comes from the later WGD (2R), whereas if the cyclostome lineage divergence postdates the duplication node, then it should be a 1R relationship. However, high G + C bias in cyclostome genomes induces significant phylogenetic challenges, meaning that the trees are often unreliable.

An alternative approach for the identification of ohnologs is based on the macrosynteny-based reconstruction of ancestral genomes (*10*, *25*, *26*, *43*). Unlike microsynteny, this strategy does not rely on gene colinearity and is less sensitive to local genomic shuffling (*26*, *31*). Given a high-resolution ancestral reconstruction and the inferred rearrangement history of chromosomes following both WGD events, it should be possible to map genomic segments onto ancestral gnathostome chromosomes and to determine whether they arose by 1R or 2R. Given that this method does not rely on cyclostome genomes, it can be combined with a phylogeny-based approach to reliably determine the timing of 1R and 2R duplicates, solving cases where only two ohnolog copies remain (Fig. 1). Such cases are intractable in isolation, but the genomic context can be informative. Furthermore, this approach may facilitate ohnolog identification in “difficult-to-solve” problems associated with hidden paralogy and inconsistent tree topologies (*44*).

In the present study, we develop an approach to ohnolog detection on the basis of integrating phylogenetics and macroevolutionary data. We present a comprehensive and high-quality ohnolog dataset distinguishing 1R and 2R paralogs that is built over an iterative process that first collects and categorizes the “easy” ohnologs (those with high sequence similarity and informative phylogenetic trees) and combines these with ancestral genome reconstructions to infer the likely WGD relationships between regions of the genome. The genomic context can then be used to reveal the relationship between “hard-to-detect” ohnologs as well as shedding light on their 1R versus 2R origins. We use this information to investigate relative contributions of 1R and 2R ohnologs to early vertebrate innovations. Furthermore, by combining multiple methods, we gain access to additional sources of evidence when calling ohnologs, leading to a higher-confidence dataset. Using this approach, we generate high-quality ohnolog datasets for 21 vertebrate species and compare them to existing work (*8*, *10*, *11*, *26*). These data are presented in an intuitive user interface at ohnologs.com, complete with extensive documentation. This allows users to easily browse, download, and perform simple analyses on our dataset.

Our approach allows us to create a comprehensive and high-quality ohnolog dataset with good coverage of available vertebrate genomes. This is a successful attempt to distinguish between 1R and 2R duplicates genome-wide, thus creating the opportunity to examine evolutionary differences between these events. This robust and thorough vertebrate ohnolog dataset, along with the website and associated analysis tools, makes our data easy to access and integrate into future work and should be a valuable community resource.

## RESULTS

### Reconstructing the ancestral vertebrate and gnathostome genomes

Macrosynteny refers to the intra- or intergenomic conservation of gene content in large chromosomal segments (*10*, *25*, *26*, *31*, *36*, *45*, *46*). The criteria are more relaxed than for microsynteny, where gene order is also considered (*11*, *47*, *48*), thus making it especially suitable to examine ancient macroevolutionary events. In particular, we wanted to delineate chromosomal segments that have remained intact (although potentially internally rearranged) since the divergence with amphioxus, allowing us to identify chromosomal fusion and fission events that occurred shortly after 1R and 2R. We identified these segments in 1 invertebrate, 6 cyclostome, and 15 gnathostome genomes by using our Bayesian segmentation model (*26*, *49*, *50*). This model detects changes in the frequency of shared homologs along a pair of chromosomes. A shift from high to low frequency of shared pairs is indicative of a macrosynteny break point, in other words, a boundary between segments originating from different ancestral chromosomes. Alternatively, unbroken segments can indicate ancestral fusion and mixing where extensive gene shuffling has led to a homogeneous distribution of homologs (*25*, *26*, *31*).

**Fig. 2.**
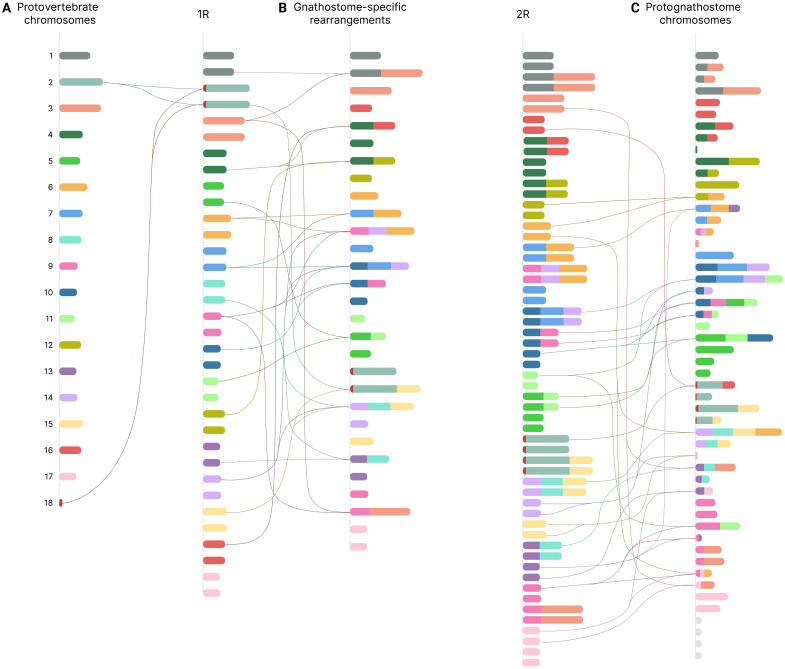
Reconstruction of the history of early vertebrate chromosomal evolution. WGD events and major chromosome rearrangements in vertebrate evolution are represented in chronological order from left to right. Each set of bars represents chromosomes at different stages of vertebrate evolution. Colors indicate the relationship to the inferred PVCs. (**A**) The protovertebrate had 18 chromosomes. (**B**) Following the first round of WGD and several chromosomal fusion events, this number increased to 29. (**C**) Last, the protognathostome had 52 chromosomes. Colored lines represent the relationships between different sets of ancestral chromosomes involving fusions and/or fission events. These are omitted if a clear relationship cannot be determined—for example, because of a lack of rearrangements—as is the case in PGCs 26 through 28. By tracking rearrangement events, we can successfully establish relationships for most of PGCs.

On the basis of our segmentation data, we used a modified version of the probabilistic macrosynteny model described in (*26*) to reconstruct (i) the pre-1R ancestral protovertebrate genome, (ii) the post-2R ancestral protognathostome genome, and (iii) the macroevolutionary chromosomal events that occurred in this time frame. Briefly, we reconstructed the ancestral vertebrate genome by arranging genomic segments into *n* protovertebrate chromosomes (PVCs) such that interchromosomal co-orthology was maximized. These PVCs are not chromosomes in the traditional sense. Instead, we define them as groups of segments from multiple species that originated from a single ancestral chromosome. Using amphioxus and cyclostome segments, we obtained a reconstruction with 18 PVCs (Fig. 2A and table S5). Similarly, we reconstructed 52 protognathostome chromosomes (PGCs) using amphioxus and gnathostome segments (Fig. 2C). These numbers are consistent with recent studies in vertebrate genome reconstruction (*25*, *26*). One indicator of the validity of these reconstructions is how they relate to the genome structure in species that were not part of the inference analysis. We found that all 18 PVCs exhibit a distinct ortholog distribution in invertebrate and gnathostome genomes that were not used in the reconstruction (fig. S4). Similarly, there is a significant enrichment of orthologs between PVCs and PGCs, often in a one-to-four relationship, as expected following two rounds of WGD (fig. S5). These observations support the validity of our reconstructions as well as their respective chromosome numbers.

### Distinguishing 1R and 2R ohnolog pairs

Early vertebrate genomes experienced two consecutive rounds of WGD, commonly labeled 1R and 2R (*21*, *22*, *25*, *26*). The 1R and 2R events are believed to be due to auto- and allotetraploidization, respectively (*26*), modes of polyploidy that are associated with different evolutionary consequences (*23*, *26*, *51*). Given the foundational timings of 1R and 2R in vertebrate and gnathostome evolution and the potential for mechanistic and evolutionary differences, there is a great value and interest in resolving the relative contributions of these two events.

**Fig. 3.**
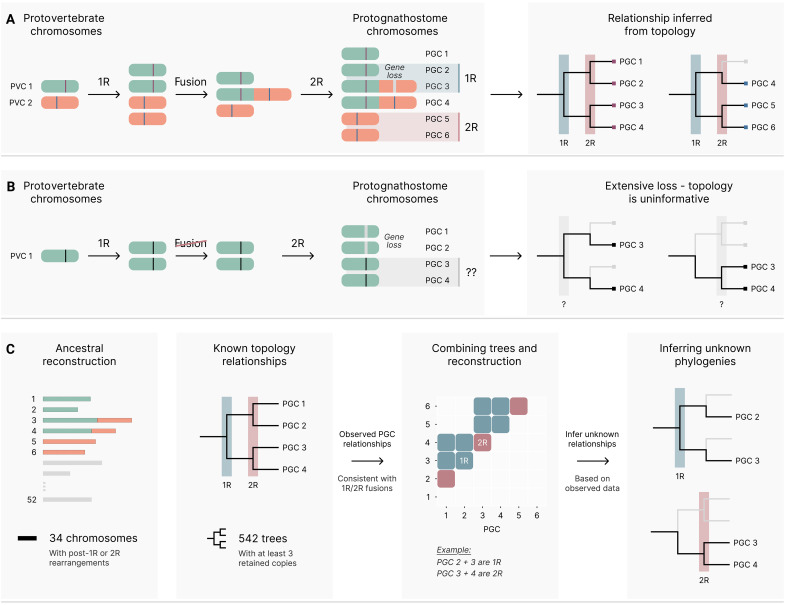
Synteny information can be informative to distinguish 1R and 2R ohnolog relationships. Colors indicate PVC relationships, and individual loci are marked with a vertical line, which is made white to indicate a gene loss. (**A**) Left: A hypothetical scenario where two PVCs are duplicated by 1R, and then one copy of each fuse before the 2R WGD duplicates everything again, resulting in six PGCs. Ohnologs will be detected across chromosome segments derived from the same PVC, as indicated by color. The timing of the chromosomal fusion in the interval between the two rounds of WGD allows us to infer that ohnolog relationships between fused and nonfused chromosomes date back to 1R, and other ohnologs across these chromosomes are related through 2R. Right: This scenario is also confirmed by gene tree topologies of ohnolog families with at least three members, showing that the earlier gene duplication relates the genes present on the fused and nonfused chromosomes. (**B**) However, ohnologs on chromosomes without telltale rearrangement events and with only two remaining members are uninformative in isolation. (**C**) We then use synteny information to extrapolate 1R/2R relationships that were inferred from telltale rearrangements or 3+ membered families to ohnologs present across the same chromosome segments. Using informative gene trees and chromosome rearrangement history, we can create a “relationship matrix” of PGC chromosomes presented as a heatmap. In the hypothetical relationship matrix shown here, a preponderance of gene trees indicates a 2R relationship between PGC 3 and PGC 4 and a 1R relationship between PGC 2 and PGC 3. We thus extrapolate this to infer that other two-membered ohnolog families shared across these chromosomes, otherwise ambiguous, share the same WGD history.

In a simple scenario where all duplicate copies have been retained, we would expect a symmetric gene tree topology showing two consecutive bifurcations. This complete phylogeny contains enough information to identify the 1R and 2R relationships on the basis of the order of events (Fig. 3A). However, it might be challenging to recover the true tree because of factors such as long branch attraction artifacts and composition bias in cyclostomes (high G + C content) (*4*, *5*, *40*, *41*), or the true gene tree might not be representative of the genome duplication timing because of processes like delayed and asynchronous rediploidization (*4*, *5*, *15*). Furthermore, in trees where only two copies have been retained, only one WGD node is present, and without further information, it is impossible to determine which WGD event is represented (Fig. 3B).

This can be largely overcome by supplementing phylogenies with synteny information (Fig. 3C) (*11*, *26*). On the basis of our reconstruction, we assigned genes to the ancestral chromosomes from which they originated. In any given tree, we expect genes related through a putative WGD node to have been assigned to different post-WGD chromosomes; otherwise, we deduce that these paralogs arose by small-scale duplication. This approach yielded 1919 trees containing 3692 ohnologs in humans. Of these, 504 trees had retained at least three copies, allowing us to determine the 1R/2R relationships of 2062 ohnologs (56% of this initial dataset) bypassing the need for a cyclostome outgroup to the 2R event. The remaining trees with only two ohnologs need further information to reliably infer the 1R versus 2R relationship.

These initial 1R/2R annotations include highly retained ohnologs only, which may plausibly have different evolutionary or biological characteristics than other, less amenable ohnologs. To relate the remaining ohnolog pairs to the 1R or 2R events, we needed to incorporate the added information from the genome reconstructions and chromosome relationships. By mapping highly retained (1R/2R informative) ohnolog pairs onto PGCs, we can infer which of these chromosomes are related by 1R or 2R on the basis of the enrichment of 1R and 2R ohnolog pairs, respectively. If we thus infer the relationship between any two PGCs, we can extrapolate this to infer the relationship between any ohnolog pair on these chromosomes. A PGC pair was deemed informative for this purpose if at least 80% of ohnolog pairs on these chromosomes originated exclusively from 1R or 2R. The chromosome pairs that passed this threshold were consistent with PGC relationships that we inferred on the basis of chromosomal rearrangements.

**Fig. 4.**
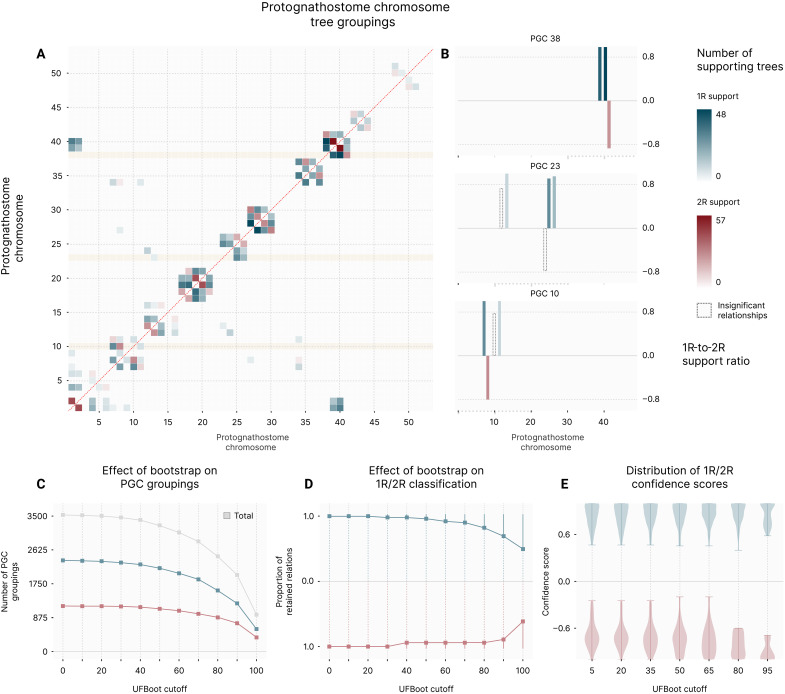
Inferring 1R versus 2R nodes for unknown trees. (**A**) Observed relationship matrix showing 1R (blue) and 2R (red) groupings for each PGC pair. The intensity of the color indicates the number of known trees that support a grouping. Only cases where at least 80% of the trees supported the relationship are shown. (**B**) Example rows from the matrix for PGC 10, PGC 33, and PGC 38 are shown, with the height of the bar indicating the proportion of trees that support either the 1R (blue) or 2R (red) topology. Relationships that favor 1R are indicated by positive values, while those that favor 2R are indicated by negative values. Pairings where <80% of trees support the relationship are not shown in (A) but are indicated here in a dotted outline. (**C**) Number of PGC groupings in informative trees that contain WGD nodes with bootstrap support at or above the given threshold indicated on the *x* axis. (**D**) Proportion of these groupings that remain at each cutoff. (**E**) Distribution of confidence scores (see Materials and Methods) for inferred PGC relationships using groupings that satisfy the given bootstrap thresholds. Positive values (shaded blue) indicate support for 1R, while negative values (shaded red) indicate support for 2R. As the bootstrap is more stringent, the distribution of values is further from zero.

If we succeeded in finding all 1R and 2R chromosome relationships, then for each PGC, we would find two PGCs related by 1R and one related by 2R. We do not find all of these for each PGC, partly due to the requirement that 80% of the informative gene trees must be concordant before we make an assignment. The 80% threshold is admittedly arbitrary and was chosen before analyzing the data as a defensible signal of data consistency. Notably, the “nonsignificant” relationships are often close to this threshold (Fig. 4B), and these could justifiably be included as the “missing” 2R or 1R relationships, although we refrain from doing that here.

Using this approach, we identified the relationships between 42 of the 52 ancestral gnathostome chromosomes. The remaining 10 PGCs did not contain sufficient 1R/2R identifiable ohnologs (i.e., <5) from our highly retained group, making any meaningful 1R/2R inference impossible. We used these relationships to build a PGC matrix that allows us to distinguish between 1R and 2R duplicates, given their ancestral chromosome assignment, without relying on phylogenetic information (Fig. 4, A and B). With this synteny information, we could classify most of our ambiguous trees: Of the 1415 two-membered ohnolog families, 389 represented a 2R pair (i.e., experienced a single loss event shortly after 1R or two convergent post-2R losses), and 314 were 1R pairs (i.e., experienced two nonconvergent loss events following 2R). The remaining 712 trees could not yet be classified.

To ensure that these relationships are not the result of phylogenetic bias, we investigated UFBoot support in 1R/2R informative trees. We found that a large proportion of inferred relationships were retained even at high UFBoot cutoffs (Fig. 4C). Furthermore, neither 1R nor 2R relationships were disproportionally affected, suggesting that these are not driven by errors and the differences observed in ohnolog retention after 1R and 2R are unlikely to be an artifact (Fig. 4D). Even at lower bootstrap cutoffs, the final confidence scores for PGC groupings are high, i.e., PGC groupings consistently favor 1R or 2R (Fig. 4E). Removing trees with low UFBoot support further increases confidence but does not result in fewer 1R and 2R ohnologs in the final dataset, illustrating that the low-support, low-confidence trees were those with other, “noisy” topologies. Furthermore, we observe concordance across synteny blocks, where the ratio of individual gene trees supporting one topology over the other is high (Fig. 4), supporting the interpretation that the synteny block represents a shared WGD history. Given that our approach relies on multiple trees to infer a true relationship, it should be robust, to some degree, to the presence of trees with low UFBoot support (see Materials and Methods).

### Set of underrepresented “hard-to-find” ohnologs

Having identified 3692 high-confidence ohnologs in humans, we set out to find any overlooked ohnologs by using additional evidence in the form of microsynteny. As distinct from the macrosynteny reconstruction, microsynteny suggests a highly conserved gene order and content (*11*, *47*, *48*). Because of these stringent criteria, any homologous blocks containing a large proportion of ohnologs are confidently inferred to have originated by WGD.

With this, we focused on two sets of candidate ohnologs: (i) putative ohnologs that could not be easily recovered by phylogenetic analysis and (ii) ohnologs that were missed because of homology detection failure (HDF) (*35*, *52*, *53*). For the former case, we found such duplicates in paired synteny blocks and ensured that each gene was assigned to a different PGC. This helped us to exclude paralogs created by segmental duplication. For the latter scenario, HDF is more likely to occur for short, fast-evolving genes with large divergence times because they are more likely to fail to pass scoring thresholds (*35*, *54*). To liberalize our sequence similarly searches to capture these HDF cases, we considered gene pairs that shared a synteny environment and that fell below our whole-genome similarity threshold but where the sequence similarity scores were significant in the smaller search space of a synteny block (a smaller search database reduces the probability of a random match). In this way, we found 100 “hard-to-find” ohnologs in humans. To verify the quality of these hits, we recalculated e-value cutoffs, taking into account the reduced search space. Of all “hard-to-find” ohnolog pairs, 82% fell below a cutoff value of 10^−4^, and all pairs fell below a cutoff value of 10^−3^, suggesting a low probability of false positives. While ohnologs are generally thought to be highly conserved (*8*, *11*, *55*, *56*), the low sequence similarity in this set potentially sets them apart and is suggestive of interesting evolutionary patterns.

### Comparison with previous datasets

**Fig. 5.**
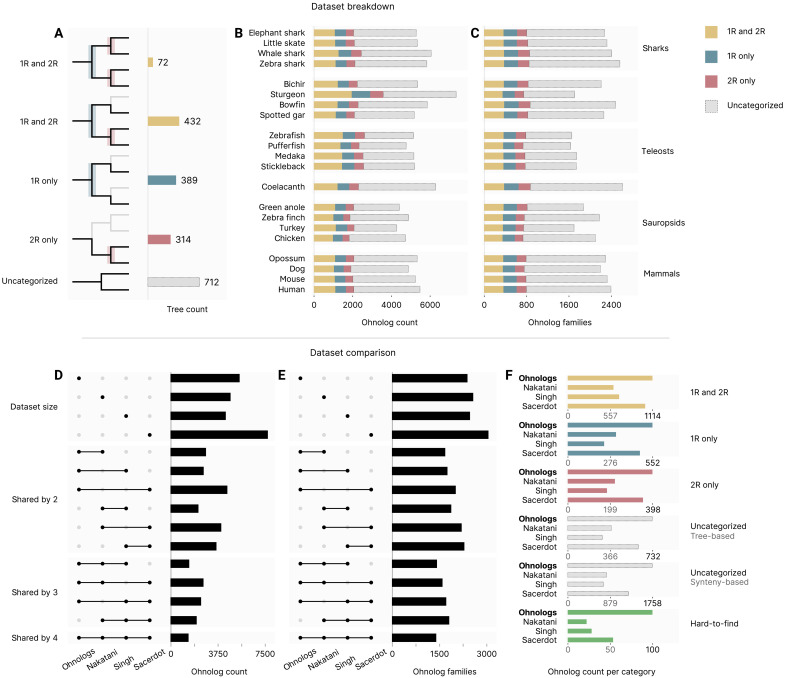
Final dataset summary. (**A**) Number of ohnolog families assigned to each 1R/2R retention category. (**B** and **C**) Total ohnolog (gene) and ohnolog family count for each species, with colors indicating the number retained after 1R only, 2R only, both WGD events, and ones that could not be categorized. (**D** to **F**) Comparison of our data (“ohnologs”) to three previously published datasets: “Nakatani” (*26*), “Singh” (*11*), and “Sacerdot” (*10*). (D) Bars showing the number of genes in common between the datasets indicated by black dots. (E) As for (D) but comparing gene families. (F) Number of ohnologs assigned to each retention category in our dataset and number of these that were found in each of the other datasets.

Our dataset contains more than 100,000 ohnologs across 21 species (Fig. 5, A to C). We found significantly more ohnologs in sturgeon (7370) than the vertebrate average (5359). This is likely the result of an extra round of WGD experienced by this lineage, although this effect is not as pronounced in teleosts despite the teleost-specific third round of WGD (Ts3R), which occurred in a similar time frame. However, both lineages could have experienced different rates of evolution and rediploidization, contributing to this effect (*16*, *24*, *57*, *58*). The lower ohnolog count in birds may be explained by the presence of microchromosomes, accounting for 33 of the 39 chromosomes in chicken (*26*, *59*). These short, gene-dense regions can be easily missed because of their length and repeat content—using modern sequencing techniques, 10 additional “dot” chromosomes have been found in the chicken genome with an average size of 3.9 Mb (*59*). However, this pattern is not observed in sharks, which also have microchromosomes. It is unclear whether these share a common origin with bird microchromosomes and, as such, could have unique characteristics, possibly with differences in ohnolog content.

Our ohnolog count in humans (5501) is comparable to that of previously published datasets (Fig. 5D), except for the study of Sacerdot *et al.* (*10*), an observation with a seemingly simple explanation: The Sacerdot dataset (*10*) is an amalgamation of datasets from multiple studies. However, this still begs the question of agreement on the labeling of ohnologs between datasets. In a two-way comparison between each study, only 42% of ohnologs appear in both sets, with only 1439 human genes labeled as ohnologs in all four studies (*10*, *11*, *26*). Similarly, only 1411 gene families contained at least one ohnolog from all four datasets (Fig. 5E). This discrepancy could have a significant impact on WGD studies. If all 9713 human genes across these datasets are real ohnologs, that would cast doubt on the accuracy of inferences about the nature of 1R/2R inferred from a small subset of these data, as most studies are. On the other hand, if there are as few as 1439 human ohnologs, the signal-to-noise ratio in current datasets is very poor, and they cannot possibly be of much use. Most likely, each dataset contains some degree of noise. While no dataset will be immune to these problems, in our case, we have openly documented the evidence base for each ohnolog assignment, and additionally, our website (ohnologs.com, more information below) allows users to select datasets and filter according to stringency and type of evidence, as befits their research question.

We also recovered 135 ohnologs in shark genomes that had reverted to a single copy in all other gnathostome genomes and were thus not flagged for analysis. These duplicates would be difficult to identify as ohnologs through phylogenetic analysis alone and were unique to our dataset. This not only demonstrates the robustness of our pipeline but may also open up the possibility of gaining insight into unique shark adaptations conferred by 1R/2R.

### Functional annotations of ohnolog subgroups

While the pivotal role of WGDs in early vertebrate evolution is well accepted, the relative or specific contribution of the 1R and 2R events has not been extensively studied, largely due to the lack of suitable data. Given that these ancient WGD events differ in timing and mechanism, it is reasonable to believe that they may have had different impacts on vertebrate genomes (*23*, *25*, *26*, *51*).

**Fig. 6.**
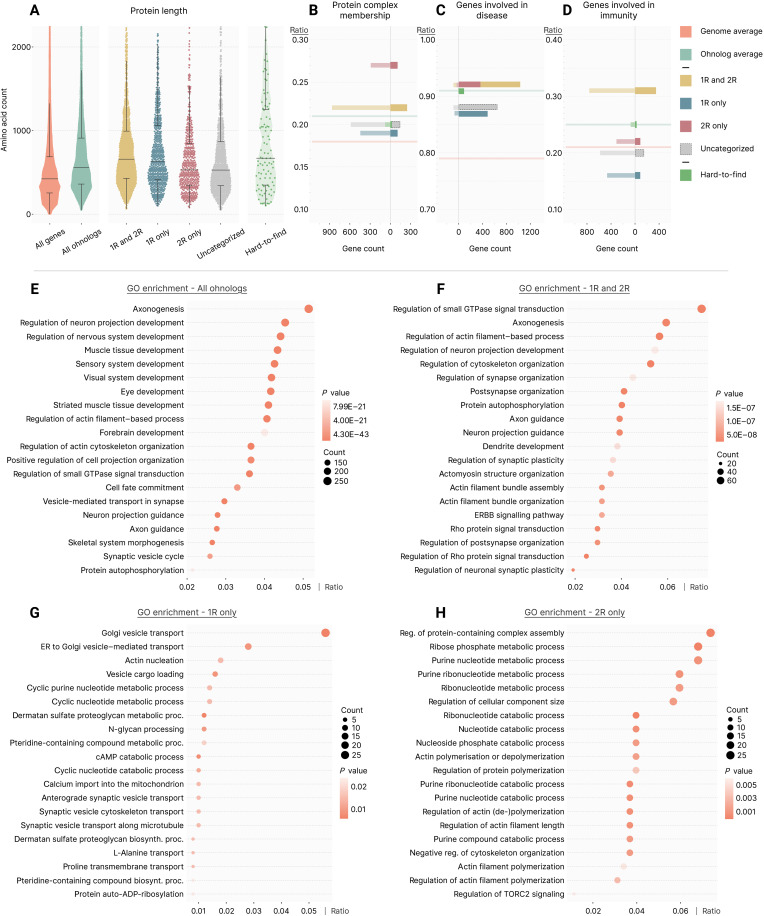
Analysis of trends in ohnolog sets. Data in (A) to (D) are colored according to the key to the right. (**A**) Distribution of protein lengths in each ohnolog category. The density of dots, representing individual ohnologs, shows the number of genes assigned to each set. (**B** to **D**) Enrichment of ohnologs in protein complexes, disease genes, and immunity-related genes. Thin orange and green lines represent the enrichment for all genes and ohnologs in the genome, respectively. Each ohnolog set is represented by horizontal bars. The length of bars (thick and thin) represents the number of genes in each category (*x* axis). The thick bar represents the number of ohnologs in the set that are enriched for each panel. The vertical position of bars represents the ratio of enriched to nonenriched genes (*y* axis). (**E** to **H**) GO term analyses for all ohnologs, ohnologs retained after both WGD events, 1R only ohnologs, and 2R only ohnologs, left to right, respectively. The proportion of enriched ohnologs is represented by the horizontal position of orange dots; the size of each dot represents the total number of genes, while the intensity of the color represents the significance of the enrichment.

We found that ohnologs were generally longer than the genomic average, confirming similar observations from previous studies (Fig. 6A) (*8*, *60*–*62*). However, duplicates retained after 2R only were significantly shorter than those retained after 1R only or after both WGD events. We are unsure of the reasons behind these differences: It could be a biological effect because of the differences between evolutionary constraints following allo- and autopolyploidy (*23*, *26*, *51*), or it could be an analysis artifact caused by increased detection failure of ohnolog relationships of short genes as the relationship gets older. Our “hard-to-find” duplicates were longer than most other ohnolog sets, suggesting that, at least for this group of genes, detection failure is presumably related to faster evolutionary rates rather than short sequence length (*35*, *54*).

Ohnologs are known to be involved in protein complexes more often than small-scale duplicates (*8*). This is what we observe in our dataset (Fig. 6B). Moreover, duplicates retained after 2R only were further enriched for protein complex membership compared to all other groups. Ohnologs have also been implicated in cancer and genetic disease (*8*, *63*). Confirming these observations, we found that our ohnologs were overrepresented in OMIM (Online Mendelian Inheritance in Man) disease genes (Fig. 6C) (*64*). Last, we examined the enrichment of genes involved in immunity (Fig. 6D) because early vertebrate WGDs have been implicated in major immune system innovations such as the evolution of the adaptive immune system (AIS) (*27*, *28*, *65*, *66*). As such, the enrichment of immune genes in our ohnolog dataset is expected. However, when ohnolog sets are examined individually, most immune-related genes are underrepresented or no different from the genomic average, except for highly retained 1R/2R duplicates. This indicates that (i) genes related to immunity were more likely to be retained after both WGD events, suggestive of adaptive benefits, and (ii) the precursors of the vertebrate AIS were likely already established before 2R (*28*, *67*). “Hard-to-find” ohnologs followed similar trends to all other groups, adding further support that these genes are ohnologs.

Our Gene Ontology (GO) term analysis indicated a bias toward development, including of sensory systems like the vertebrate-specific camera-type eye, and cell-cell signaling in all ohnologs (Fig. 6E), consistent with previous work (*7*–*9*, *11*) and thus lending credibility to our dataset. Ohnologs retained after both WGDs were much alike, with an emphasis on neuron and cytoskeleton function (Fig. 6F). Duplicates retained after 1R were uniquely enriched for cellular transport pathways (Fig. 6G), while those retained after 2R were enriched for nucleotide processing (Fig. 6H). Unclassified ohnologs appeared as a mixture of all previous sets, providing additional evidence for their classification as ohnologs. This set was also enriched for autophagosome-related pathways (fig. S1). Autophagy is involved in many cellular housekeeping, homeostasis, and resourcing processes (*68*) including adaptive immunity where it is involved in the development and survival of lymphocytes as well as the modulation of antigen processing and presentation (*69*–*71*), thus providing an additional link between 1R/2R and adaptive immunity. Our “hard-to-find” duplicates were uniquely enriched for mRNA processing and kidney development (fig. S3). Kidneys are thought to have first appeared in vertebrates and to have facilitated adaptation to different environmental conditions through continued innovations throughout vertebrate evolution (*72*). These characteristics may contribute to the presumably fast evolutionary rate of this group.

### Investigating ohnolog retention patterns

**Fig. 7.**
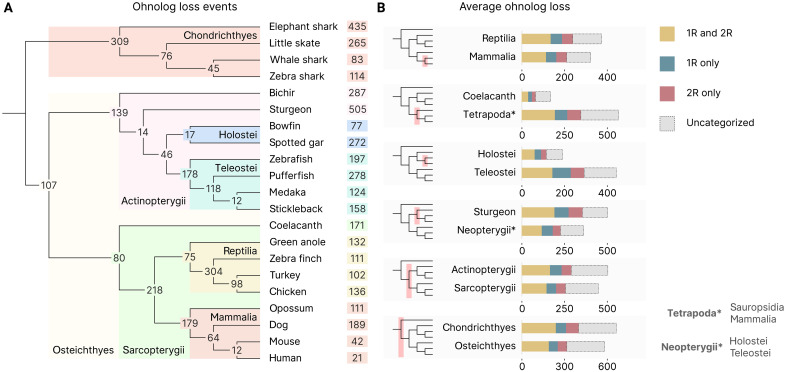
Ohnolog loss events. (**A**) Species tree of the animals included in this study, with major clades shaded and labeled. Branch lengths are not to scale. Each node is labeled with the number of ohnolog loss events occurring at that point, with lineage-specific losses indicated beside the species names. (**B**) A simplified phylogenetic tree, showing only the major clades [as per the shading in the tree in (A)], is presented to the left of each panel. To the right is the comparison of average ohnolog loss rates (see Materials and Methods) between sister clades separated by the highlighted branching point. The numbers of loss events are divided into 1R/2R retention categories.

We briefly investigated ohnolog retention patterns in our dataset (Fig. 7). While ohnologs are known to be highly conserved (*8*, *11*, *55*, *56*), ohnolog loss still occurs in all lineages. While we cannot exclude homology detection failure (HDF), which may affect some parts of the tree more than others, we nonetheless explore the patterns of ohnolog absence. We find that the ohnolog absence is generally consistent between sister clades and appears to be balanced between 1R/2R retention categories. Furthermore, we observe many putative loss events across vertebrate lineages, suggesting that ohnolog loss has been a consistent occurrence throughout vertebrate evolution. In effect, ohnologs in distantly related lineages may belong to different retention categories. These would presumably be under different evolutionary constraints, which could lead to the evolution of unique traits in each lineage.

However, not all lineages follow the same ohnolog retention patterns. Teleost and sturgeon genomes experience an elevated amount of loss, possibly the result of additional rounds of WGD experienced by these lineages. The additional copies presumably confer increased plasticity to previously duplicated gene families (*16*, *24*, *57*, *58*). Conversely, we observe a reduced amount of loss in coelacanths. This is unlikely to be the effect of low-quality genome data, as this would instead result in a higher perceived amount of loss. Coelacanths, dubbed “living fossils,” are known for their incredibly slow rate of evolution (*73*), making it tempting to suggest that this explains the lower ohnolog loss observed for this species. However, other substantially slowly evolving “living-fossil” species show relatively high ohnolog loss (e.g., elephant shark, spotted gar, and sturgeon; Fig. 7), controverting the idea of a correlation between the rate of molecular evolution and the rate of ohnolog loss in jawed vertebrates.

### User-friendly, modern web interface

**Fig. 8.**
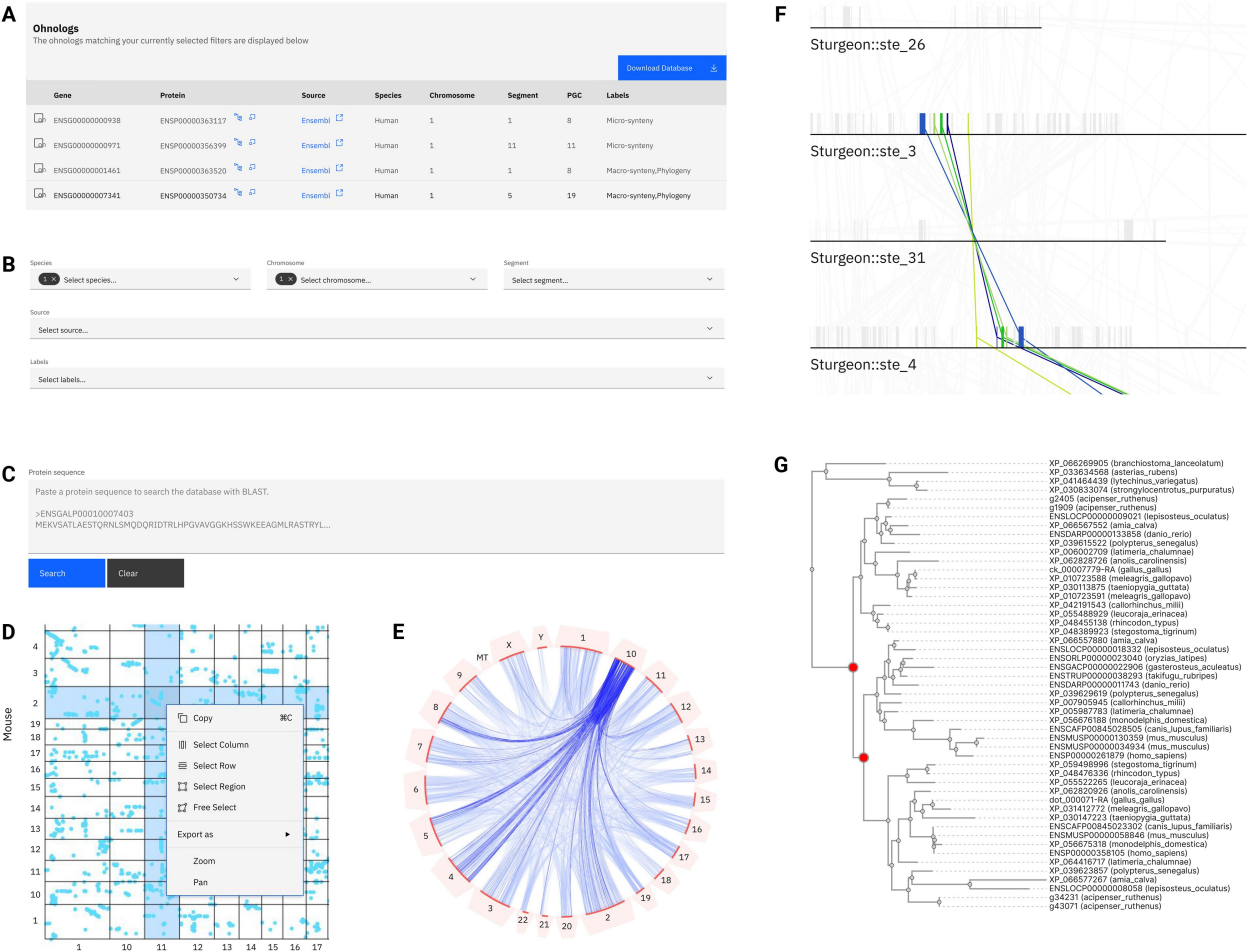
Ohnologs.com website. A selection of graphical, analytical, and data access features of ohnologs.com is illustrated here with screenshots from the website. (**A**) All the ohnologs in our database are displayed in tables. These can be downloaded, filtered, or viewed in other databases. (**B**) Users can select various filters to choose which genes to display. (**C**) Our database can be searched by protein sequence using BLAST. (**D**) Dot plot of ohnolog relationships across two genomes. Users can interactively select different regions of the plot, and further details of the genes in the selected region will be provided. (**E**) Circos plot of intragenomic ohnolog relationships. The relationships involving chromosome 10 are highlighted for illustration. Users can highlight ohnologous regions of chromosomes of their choice. (**F**) Microsynteny plot displaying ohnolog relationships across the chromosomal regions illustrated. Selecting a gene will display all of its homologs. (**G**) Gene tree of a multigene family. WGD nodes are highlighted.

We live in an always-online world—every device and individual are connected. Of course, bioinformatics software has taken advantage of this: Services such as BLAST from National Center for Biotechnology Information (*74*) or ITOL provided by European Molecular Biology Laboratory (*75*) make routine data analysis simple and accessible. In this spirit, we aim to provide a comprehensive suite of online tools that can be used for preliminary analyses of ohnologs (Fig. 8).

We offer several convenient ways to search our database. A list of all ohnologs and species contained in our dataset can be found at https://ohnologs.com/ (Fig. 8A). This includes metadata such as genome source, version, and gene count. Each of these can be filtered, allowing users to analyze and download their preferred subsets of genes (Fig. 8B). Alternatively, users can provide their own preferred lists of gene or protein identifiers in FASTA, TSV, or CSV format. Any dataset entries matching these will be displayed and can be downloaded. Database searches can also be conducted using a protein sequence on the BLAST page. The speed improvements offered by DIAMOND (*76*) allow us to provide search results in real time (Fig. 8C).

We offer a comprehensive suite of tools, allowing users to visualize and interact with our data in various ways. Circos plots can be generated for each genome in our dataset, providing an easy method to identify intraspecies WGD blocks (Fig. 8D). Similarly, our dot plot utility helps identify blocks of macro- or microsynteny between chromosomal regions. By right-clicking on the plot, users can select individual chromosomes or regions of interest. These will then be highlighted and can be downloaded for further analysis (Fig. 8E). In-depth visualizations of microsynteny blocks featured in our analysis are available on the synteny page. These plots contain many interactive elements. Clicking a gene will highlight all of its homologs, while hovering over it with the mouse cursor will display additional information (Fig. 8F). Similarly, all gene trees used in our analysis can be viewed on their corresponding page (Fig. 8G).

Each plot is accompanied by a table listing all relevant entries from our dataset. Any gene selected in the table will be highlighted in the plot above. Similarly, any gene selected through the plot will be highlighted in the table. This “two-way selection” allows users to visually identify points of interest and download the corresponding data for downstream analysis. This selection is persistent across pages, allowing cross-referencing of multiple plots.

Several workflows may particularly benefit from ohnologs.com. Users with a favorite gene or gene family can use our BLAST utility to identify ohnologs of interest, adding any hits to their selection. These genes will then be highlighted in all tables and figures across our website. For more complicated use cases, we offer comprehensive documentation detailing every aspect of our tooling. Web services, or any tools for that matter, are completely useless if they are without instructions—even furniture comes with a how-to guide these days! Lest we are outdone by IKEA, we offer simple guides at the top of each page, as well as more detailed instructions for advanced users, hosted at https://docs.ohnologs.com/. This website contains detailed information about our dataset, each page on our website, file formats for dataset downloads, and power-user features.

A major appeal of web-based databases is the accessibility they offer, providing the same services to all individuals, regardless of personal capabilities or circumstances. We believe that science should be open to all and so have endeavored to make our website accessible to a wide range of users; All of our content, be it tables or figures, is responsive and should function on any device—a phone, tablet, laptop, or desktop. We use HTML semantics and ARIA (accessible rich internet applications) attributes to make our interface accessible to those with disabilities. This includes utilities such as image alt-text and a tab index, allowing for navigation without a mouse—essential features for those with screen readers. Similarly, the typography and colors on our website were selected with visually impaired users in mind. In short, we have made an effort to comply with the web content accessibility guidelines (WCAG) 2.0 specification. Last, in the spirit of openness and inclusivity, https://ohnologs.com/ is licensed under GPLv3—anyone is free to adapt our software.

## DISCUSSION

Previous attempts at creating comprehensive 1R/2R ohnolog datasets have been inconsistent, resulting in highly variable annotations with as few as 1144 human ohnologs in common. This poses an important question—how many “ohnologs” are actually ohnologs? By developing a novel technique and combining multiple sources of evidence, we were able to avoid the common pitfalls associated with previously used methods (*8*, *10*, *11*, *26*). Our dataset is comprehensive, has high quality, and covers a large number of available vertebrate genomes.

A core part of our approach, and major distinguishing feature, is our reliance on state-of-the-art ancestral genome reconstructions (*26*). Here, we provide a high-resolution reconstruction of the ancestral vertebrate and gnathostome genomes. While these have been attempted before, the number of ancestral vertebrate chromosomes has been contentious (*10*, *25*, *26*). We provide additional evidence for 18 chromosomes in the protovertebrate ancestor, first proposed in (*26*). Our work also demonstrates that ancestral reconstruction is a robust and reliable way to supplement phylogenetic trees in difficult-to-solve problems where large amounts of error are expected—in this case, because of composition bias and long-branch attraction artifacts caused by cyclostome genomes (*4*, *5*, *40*, *41*). Similar methods may help resolve difficult questions surrounding 1R/2R, such as delayed rediploidization in crown vertebrates and cyclostomes (*4*, *5*).

Our modern online interface offers major innovations over its predecessors (*11*, *77*) because it uses the latest web technologies, provides broad functionality, and is fast. We believe that genomic data are only useful if they are easy to access and well documented. As such, our online interface allows users to browse, visualize, and perform simple analyses on our dataset. This should be especially useful for those interested in a specific gene or gene family. In the spirit of accessibility, we provide tutorials explaining common usage patterns in our online usage manual. We endeavor to expand this resource in the future to include ohnologs from other vertebrate WGDs (*16*, *23*, *24*, *51*, *78*), together with additional genomic information such as gene expression data (*79*). This, together with an easy way to integrate with well-established databases such as Ensembl and RefSeq (*80*, *81*), will allow us to build a suite of tools for powerful comparative genomic analyses. Our efforts should pave the way for and simplify further analyses involving these ancient WGDs.

Our dataset differentiates between 1R and 2R duplicate pairs on a genome-wide scale. These successive WGD events are thought to have occurred through different mechanisms, auto- and allopolyploidy, respectively, the nature of which has a notable effect on genome evolution (*23*, *26*, *51*). After allopolyploidy, as observed in 2R, one of the duplicated genomes often experiences a higher amount of gene loss (*25*, *26*). This phenomenon has been well characterized in plants, with recent additional examples in vertebrates, and is thought to be caused by incompatibilities between protein interaction networks and differences in expression between genomes (*23*, *82*). Our annotation allows us to delve deeper into this aspect of early vertebrate evolution, which has not seen much attention before.

There are apparent functional differences between 1R and 2R duplicates in areas such as protein complex membership, disease, and immunity. While 1R duplicates are more involved in cellular transport pathways, those retained after 2R are highly enriched for nucleotide processing. However, the most notable difference lies in immunity—only duplicates retained after both 1R and 2R are enriched for immune genes. It is widely accepted that 1R and 2R were instrumental in the evolution of the AIS, the cellular precursors of which are thought to have existed in the vertebrate ancestor (*67*, *83*). However, our data suggest that both 1R and 2R have played an important role in shaping the modern AIS, elaborating on any components that may have already been present in a stepwise manner rather than a single “big bang.” As 1R is shared by all vertebrates, this might also explain why adaptive immunity in cyclostomes is accomplished through genetically distinct immune receptors while still sharing many components with the gnathostome AIS (*28*, *40*, *67*).

We also define a previously underrepresented class of “hard-to-find” ohnologs that did not meet initial sequence similarity thresholds. These were likely missed in previous studies because of HDF, which has been demonstrated in multiple other clades (*35*, *52*, *53*). HDF occurs because of the saturation of sequence information, which is especially prevalent in short and fast-evolving genes (*35*, *54*). Our set of “hard-to-find” ohnologs is functionally distinct from any other group and goes against our established understanding of human ohnologs as being highly conserved and slowly evolving (*8*, *11*, *55*, *56*). It is now unclear whether these genes, like many other ohnologs, are uniquely duplicable by WGD or whether they are generally highly duplicable. Because they are hard to detect, we cannot rely on sequence similarity to answer this question, which complicates most conventional analyses.

Just as delineating 1R and 2R ohnologs reveals a role for genome duplication in the formation of adaptive immunity and other aspects of vertebrate biology, identifying these “hard-to-find” ohnologs provides insights into vertebrate evolution and innovations. For example, “hard-to-find” ohnologs are implicated in the development of the kidney, an organ that first appeared in the vertebrate ancestor having transitioned from invertebrate metanephridia (*84*). Similarly, our dataset as a whole is strongly consistent with a role for ohnologs in the development of vertebrate sensory systems, including the camera-type eye that emerged in the vertebrate ancestor (*85*, *86*). Alongside these insights into major vertebrate innovations, our results indicate that ohnologs are vital cogs in the cellular biology of vertebrates, influencing protein complexes, cell signaling, and communication. This clearly indicates that the two rounds of genome duplication have not only driven genetic distinction from invertebrates but also bestowed vertebrates with notably unique cell biology, perhaps one of the most extraordinary vertebrate novelties of all.

## MATERIALS AND METHODS

### Dataset and quality control

We obtained the 36 proteomes used in this study from various sources (see table S1). We used OrthoFinder (2.5.4) (*87*) to identify orthogroups and run an all-versus-all similarity search using DIAMOND (2.0.14) (*76*). The DIAMOND run was performed in ultrasensitive mode with the --ultra-sensitive flag. To split hierarchical orthogroups and stop the analysis after orthogroup inference, the -og and -y flags were used respectively in OrthoFinder.

We used M-Coffee (*88*) to identify which, if any, orthogroup sequences could not be aligned consistently. We used the following alignment software: MAFFT (version 7.490) (*89*), MUSCLE (5.0.1428) (*90*), Clustal Omega (1.2.4) (*91*), and T-Coffee (13.45.58.c355d11) (*92*). These were selected to provide a good sampling of different alignment methods (*88*, *93*). Sequences were removed if less than 40% of their length could be aligned reliably. We used GUIDANCE (2.02) (*94*) to identify and remove alignment positions that are not robust to the guide tree used to build the alignment, as such positions can mislead phylogenetic inference. Positions with a GUIDANCE confidence score of less than 93% were trimmed. The remaining sequences were aligned using MAFFT, using the --maxiterate 1000 and --localpair flags.

### Homology inference

We used the all-versus-all DIAMOND results generated by OrthoFinder to build ortholog sets for our pipeline. For each query gene, we defined its orthologs as the top *n* best scoring DIAMOND hits, where *n* is based on the number of WGDs separating the query and subject species (see multiplicity in table S2). For example, we found 1:1 orthologs between humans and mice as their genomes share the same number of WGD events. However, compared to amphioxus, the human genome has experienced two additional WGD events. As a result, we found 1:4 orthologs between these genomes.

We generated an initial ohnolog set required for our ancestral reconstructions by identifying paralogs whose duplication timing coincides with that of the 1R/2R WGD events. Given two reference species, a pair of genes with a bitscore s was annotated as a paralog pair if they met the following conditions: (i) The shorter gene does not have a larger than s bitscore to any genes from genome A. b) The shorter gene has a larger than s bitscore to the best-matching gene from genome B. Genome trios for this analysis are shown in table S2. To exclude large gene families, we discarded the gene pair if it was not one of the top *n* best scoring DIAMOND hits. The value of *n* was determined by the number of expected duplicates given the WGD history of a species (see table S2). In addition, paralogs located within five genes of each other on the same chromosome were discarded.

### Ancestral genome reconstruction

We developed a Bayesian sequence segmentation algorithm to identify genomic regions of conserved macrosynteny (*43*, *49*, *50*). Macrosynteny can be defined as the conservation of gene content between homologous chromosomal regions. We can identify the boundaries (hereafter break points) separating these regions on some chromosome Q by identifying changes in the frequency of homologs along the chromosome when compared to some other chromosome S. Briefly, the model works as follows: Let *R* be the observed sequence of genes along chromosome Q. These genes are split into two categories *c* on the basis of their similarity to genes on some other chromosome S: *c* = 0 in the absence of any similarity and *c* = 1 if the gene has at least one homolog on S. Given κ, the number of synteny break points in sequence R, the probability of observing such a sequence is given as follows

$$P\left(R\right)=\sum_{k=0}^{k_{\text{max}}}P\left(\kappa =k\right)P\left(R|\kappa =k\right)$$ (1)

We can then calculate P(κ=k)P(R∣κ=k) given the formula below, where *A* represents the segmentation of sequence *R*, referring to the number and position of synteny break points, $n_{k}$ is the number of genes in segment *k*, $n_{k,c}$ is the number of genes in segment *k* that belong to category *c*, and $\alpha_{c}$ is the prior for category *c*

$$P\left(R|\kappa =k\right)=\sum_{A}\prod_{k}\frac{\Gamma \left(\Sigma_{c}\alpha_{c}\right)\Pi_{c}\Gamma \left(n_{k,c}+\alpha_{c}\right)}{\Pi_{c}\Gamma \left(\alpha_{c}\right)\Gamma \left(n_{k}+\Sigma_{c}\alpha_{c}\right)}$$ (2)

This allows us to calculate the number of break points in the chromosome up to some value $k_{\text{max}}$. Given that the model is effectively intractable for values of *k* > 2 using a brute-force approach, we used dynamic programming to complete the calculation. Once we know the number of break points in a chromosome, we can work backward to find the locations of these break points. A detailed explanation and a reference implementation in Python can be found on GitHub. The implementation used in our analyses was written in Rust and called using Python FFI, significantly increasing the performance of our pipeline.

We used the following parameters for all comparisons: *k* = 20, α = 1.0, and β = 1.0. For the protovertebrate reconstruction, amphioxus and six cyclostome genomes (table S3) were segmented against two sets of genomes. The first set contained sea lamprey and inshore hagfish. The second set contained chicken, turkey, opossum, spotted gar, and elephant shark. Postprocessing steps were applied to the resulting break points. Break points were kept if at least *n* break points appeared within a window of five genes. The value of *n* is equal to the number of query genomes in each group. Similarly, amphioxus and 15 gnathostome genomes (table S4) were segmented for the protognathostome reconstruction. Again, the query genomes were divided into two groups: Group one contained chicken, turkey, opossum, spotted gar, and elephant shark. Group two contained zebrafish, medaka, stickleback, and pufferfish. The same postprocessing steps were applied with different values of *n*. The value of *n* was determined as follows: For the first group, this is equal to the number of genomes in the group. For the second group, this is equal to twice the number of genomes in the group. This value reflects the difference in the number of WGD events separating the reference and query genomes (see table S3 for summary).

A modified version of the reconstruction algorithm from (*26*) was used to reconstruct the ancestral vertebrate and gnathostome genomes. We used amphioxus and six cyclostome genomes for the protovertebrate reconstruction (table S3). The amphioxus genome was used as a reference. Default parameters were used unless specified otherwise. The reconstruction was performed with values of *K* ranging from 10 to 24, and the most significant reconstruction (table S5) was selected using a hypergeometric test. Let *g* and *p* be the number of gene and paralog pairs in the genome, respectively. Let *v* be the number of gene pairs that both derive from the same PVC. Let *X* be the number of paralog pairs that both derive from the same PVC and *x* be the observed number of such pairs. The significance of reconstruction, *x*, is given as follows

$$P\left(X\geq x\right)=\sum_{i=x}^{v}\left(\begin{array}{c} p-i\\ g-v \end{array}\right)\left(\begin{array}{c} i\\ v \end{array}\right)/\left(\begin{array}{c} p\\ g \end{array}\right)$$ (3)

We used amphioxus and 15 gnathostome genomes (table S4) for the protognathostome reconstruction. We first reconstructed ancestral vertebrate linkage groups in a similar manner to the protovertebrate reconstruction. This reconstruction was performed for values of *K* ranging from 6 to 20, and the most significant reconstruction (table S6) was selected according to Eq. 3. The number of linkage groups was lower than the predicted number of ancestral vertebrate chromosomes, likely due to extensive chromosomal fusions and gene shuffling events experienced by gnathostome genomes (*26*). On the basis of this, we reconstructed PGCs by clustering the genomic segments assigned to each vertebrate linkage group using methods developed by previous work (*26*).

We identified chromosomal rearrangements (i) between the 1R and 2R events and (ii) following both WGD events. To identify ancestral chromosome pairs that share a significant number of orthologs, we used the Poisson distribution, assuming that orthologs are randomly distributed. Relationships were classified as significant where *P* < 10^−2^. The ancestral vertebrate genome was represented by Japanese lamprey genes. We performed this analysis for every genome in the protognathostome reconstruction, and relationships were kept if they appeared in at least three species. Rearrangements were classified as having occurred between the two WGD events if at least two PGCs shared synteny with two or more PVCs. Otherwise, we assumed that the rearrangement occurred after the 2R event.

### Phylogenetic analysis

We used IQ-TREE (2.1.3 COVID-edition) (*95*) for building gene trees. We used the following parameters: -bb 1000 - creates 1000 bootstrap replicates (*96*). -bnni - optimizes bootstrap trees to prevent overestimating branch supports resulting from severe model violations. -allnni - enables a more thorough nearest-neighbor interchange search. -m JTT+G - uses the given model for tree building. This model has been used in previous analyses investigating ancestral gnathostome WGD events (*16*). Cyclostome genomes were excluded from the phylogenetic analysis because of their fast evolution and composition bias.

We used the ETE-3 Python library (3.1.2) (*97*) for our phylogenetic analysis. Gene trees were rooted on acorn worm or amphioxus if there were no acorn worm genes. Any trees without invertebrate genes or trees that could not be rooted on a single node were discarded. If an early duplication event before the divergence of amphioxus could be inferred, such trees were split into two subtrees for the remainder of the analysis. Ohnologs (or the lack thereof) in each tree were found as follows: All genes in a tree were annotated on the basis of their placement on PGCs. Given the nature of the 1R/2R events, for any two given PGC annotations, a and b, the latest common ancestor (LCA) of a should never contain b as one of its children, and vice versa. Such a situation could only arise because of errors in our reconstruction or tree-building steps. Therefore, these annotations were discarded. For each remaining PGC represented in the tree, its LCA node was found such that all genes belonging to some LCA node n had been placed on a unique PGC in our reconstruction. LCA nodes were discarded if they contained (i) fewer than three genes, (ii) fewer than two PGC annotations, or (iii) genes from a single species or (iv) more than half of the PGC annotations were discarded in earlier steps.

The topology of the remaining LCA nodes was used to determine the number and type of WGD events: (i) Genes in trees with one LCA node have not retained any duplicate copies following WGD. (ii) Genes in trees with two LCA nodes have retained two of the four copies following two rounds of WGD, but we cannot determine which copies have been retained using the tree topology. (iii) Genes in trees with three or four LCA nodes have retained enough copies that we can determine whether they were duplicated by 1R or 2R by investigating the tree topology.

We know that LCA nodes originating from 2R should be more closely related to each other than to any other LCA node in the tree. We used the topology of unambiguous trees where at least three of the four ohnolog copies had been retained to see (i) if our data followed this pattern and (ii) if it was consistent with the PGC relationships from our reconstruction. For all of the 1R and 2R relationships of some LCA node a, if another node b appeared in at least 80% of those 1R or 2R relationships, these LCAs were deemed to be grouping consistently. These consistent groupings were then used to infer whether duplicates in ambiguous trees were related by 1R or 2R.

### Microsynteny analysis

We used i-ADHoRe (version 3.0) (*47*) to identify blocks of conserved microsynteny between gnathostome genomes. We used a Python script to (i) deduplicate the hits produced by i-ADHoRe and (ii) cluster all syntenic segments into multispecies blocks. We identified additional ohnologs as follows: (i) We removed any multispecies blocks that contained less than five genes or those in which less than 20% of genes were tagged as ohnologs on the basis of our gene tree analysis. (ii) In the remaining blocks, we identified paralogs that were assigned to different PGCs, ensuring that they were not created by segmental duplication. (iii) These hits were split into regular (*E* < = 10^−4^) and “hard-to-find” (0.05 > = *E* > 10^−4^) ohnologs. (iv) For genomes that were not used in the reconstruction and so did not have PGC assignments, we classified paralogous pairs as ohnologs if they were members of a gene family that contained other previously identified ohnologs.

### Calculating ohnolog loss rates

We identified WGD nodes in 1919 trees and dated gene loss events using the ETE-3 Python library (3.1.2) (*97*). Average loss for a clade can be calculated as follows, where *n* is the current node, and *c*1/*c*2 are children of *n*

$${\text{Average loss}}_{n}={\text{Observed loss}}_{n}+\left({\text{Average loss}}_{c1}+{\text{Average loss}}_{c2}\right)/2$$ (4)

Observed loss represents the counts obtained from dating gene loss events and is equal to average loss for leaf nodes. For example, the average loss for the common ancestor of mice and humans can be expressed as 12 + (42 + 21)/2 (see Fig. 7). Loss counts may be biased by GUIDANCE and T-Coffee trimming performed in our pipeline. However, this should not have a large effect on our results, assuming that trimming was not biased by species. Our lineage sampling could also be a source of bias as some genomes, which may experience faster or slower rates of loss, could be under- or overrepresented.

### Building our web interface

We used SvelteKit with the Carbon Components UI library to build our interface. Data visualizations were designed from scratch using SVG elements with the help of D3, a data visualization library for JavaScript. Our gene trees were rendered using a third-party “phylotree” layout (*98*). Our dataset is stored in a PostgreSQL database and queried using Prisma. We use GitHub Actions to run automated code quality checks and provide up-to-date builds in the form of Docker containers. Our network stack consists of Cloudflare DNS and an Nginx reverse proxy. All connections are secured with TLS.
