# Supplementary material for: A comprehensive and accessible database of jawed-vertebrate ohnologs
Source: Sci Adv. 2026 Aug 7;12(32):eadz8270. doi: 10.1126/sciadv.adz8270 (PMC13450315; doi:10.1126/sciadv.adz8270)
Supplement: Supplementary file 1 [file sciadv.adz8270_sm.pdf]

Science Advances
AAAS

Supplementary Materials for

A comprehensive and accessible database of jawed-vertebrate ohnologs

Łukasz Niezabitowski *et al.*

Corresponding author: Aoife McLysaght, aoife.mclysaght@tcd.ie

Sci. Adv. **12**, eadz8270 (2026)
DOI: 10.1126/sciadv.adz8270

This PDF file includes:

Figs. S1 to S5
Tables S1 to S6

Supplementary information for: A comprehensive and accessible database of jawed-vertebrate ohnologs

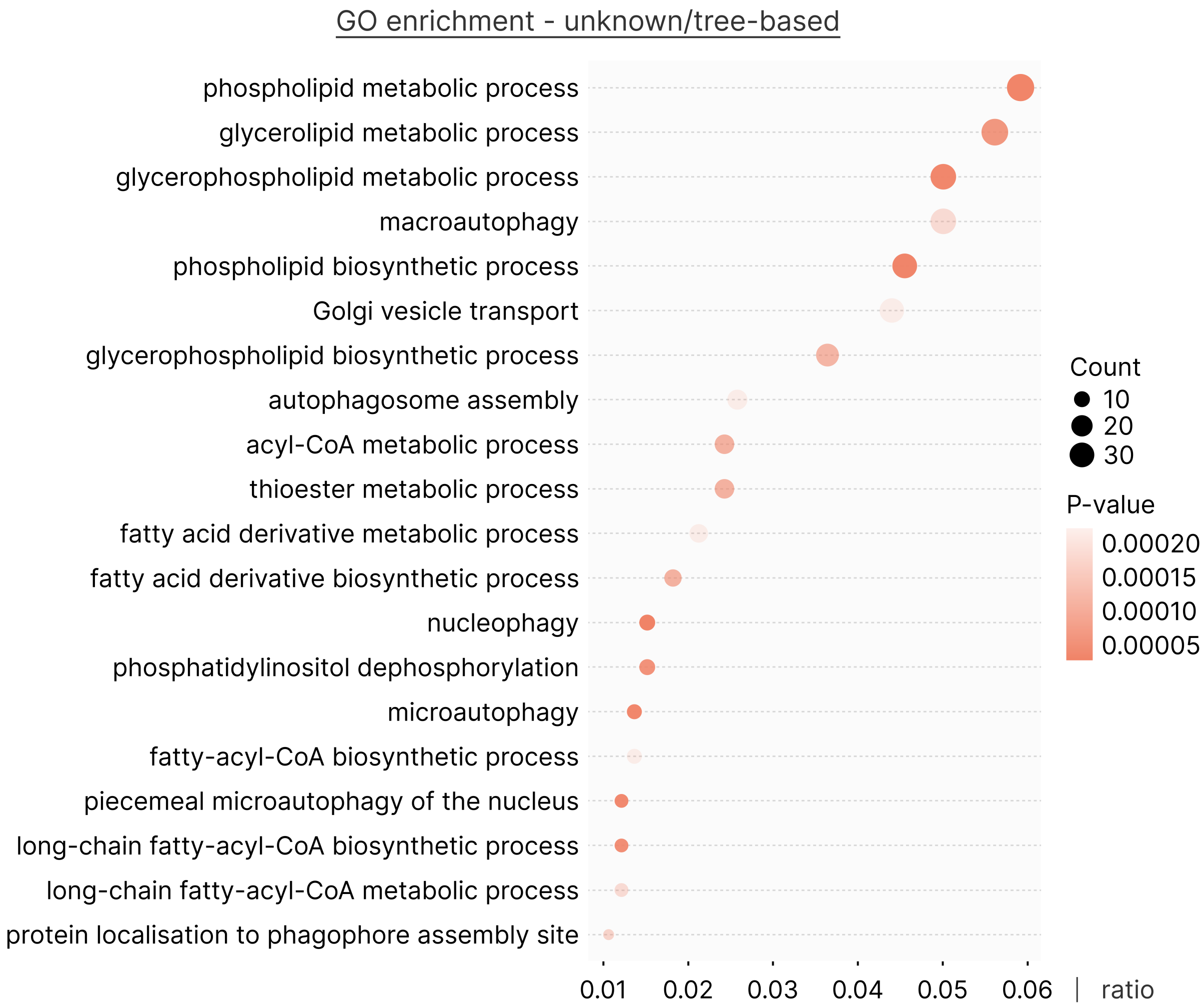

Fig. S1. GO term analysis for tree-based ohnologs in the unknown retention category. This set includes ohnologs from the gene tree analysis that could not be confidently classified as retained after 1R only or 2R only.

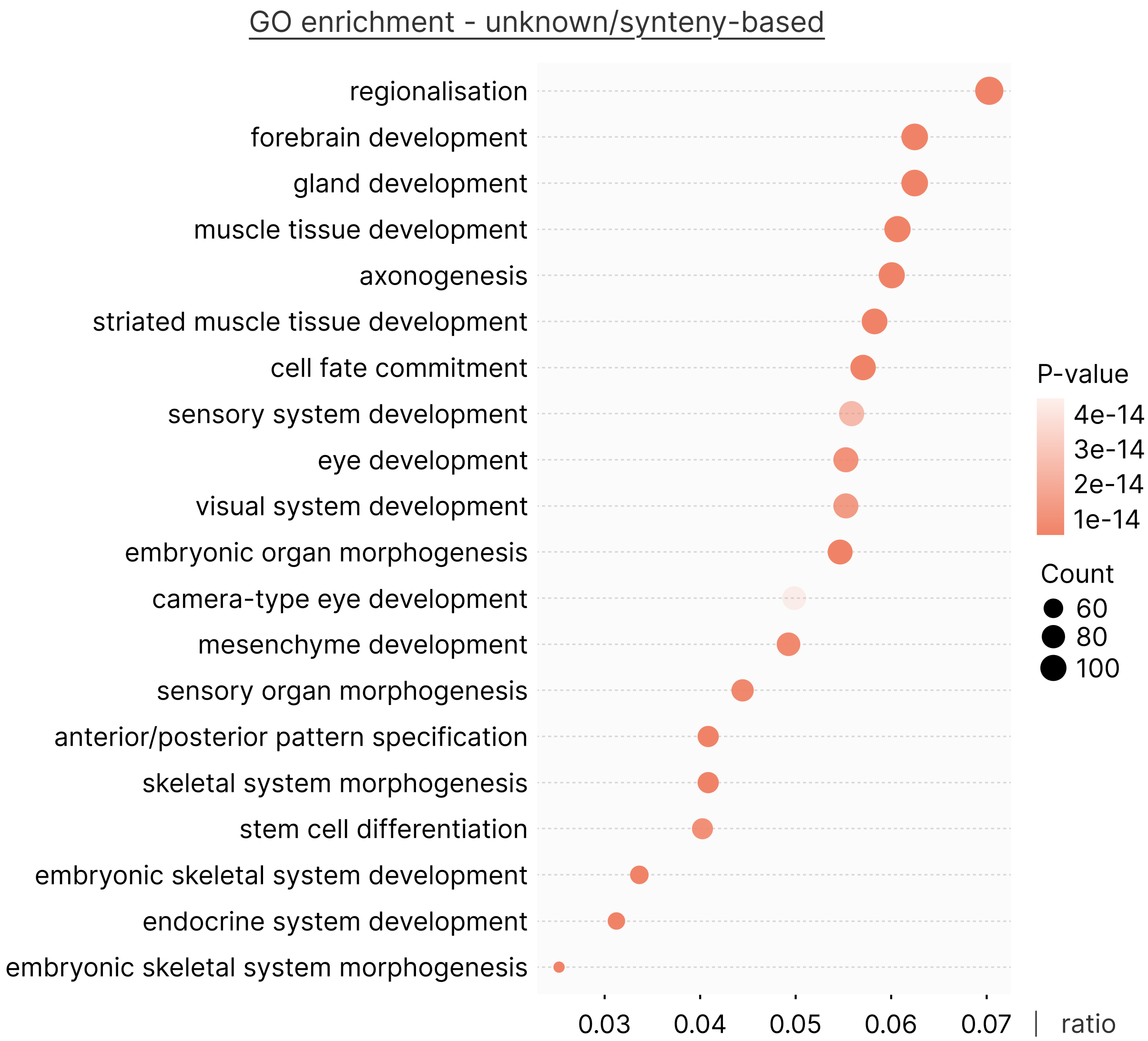

Fig. S2. GO term analysis for synteny-based ohnologs in the unknown retention category. This set includes ohnologs from the microsynteny analysis. This set cannot be classified based on retention patters due to a lack of phylogenetic information.

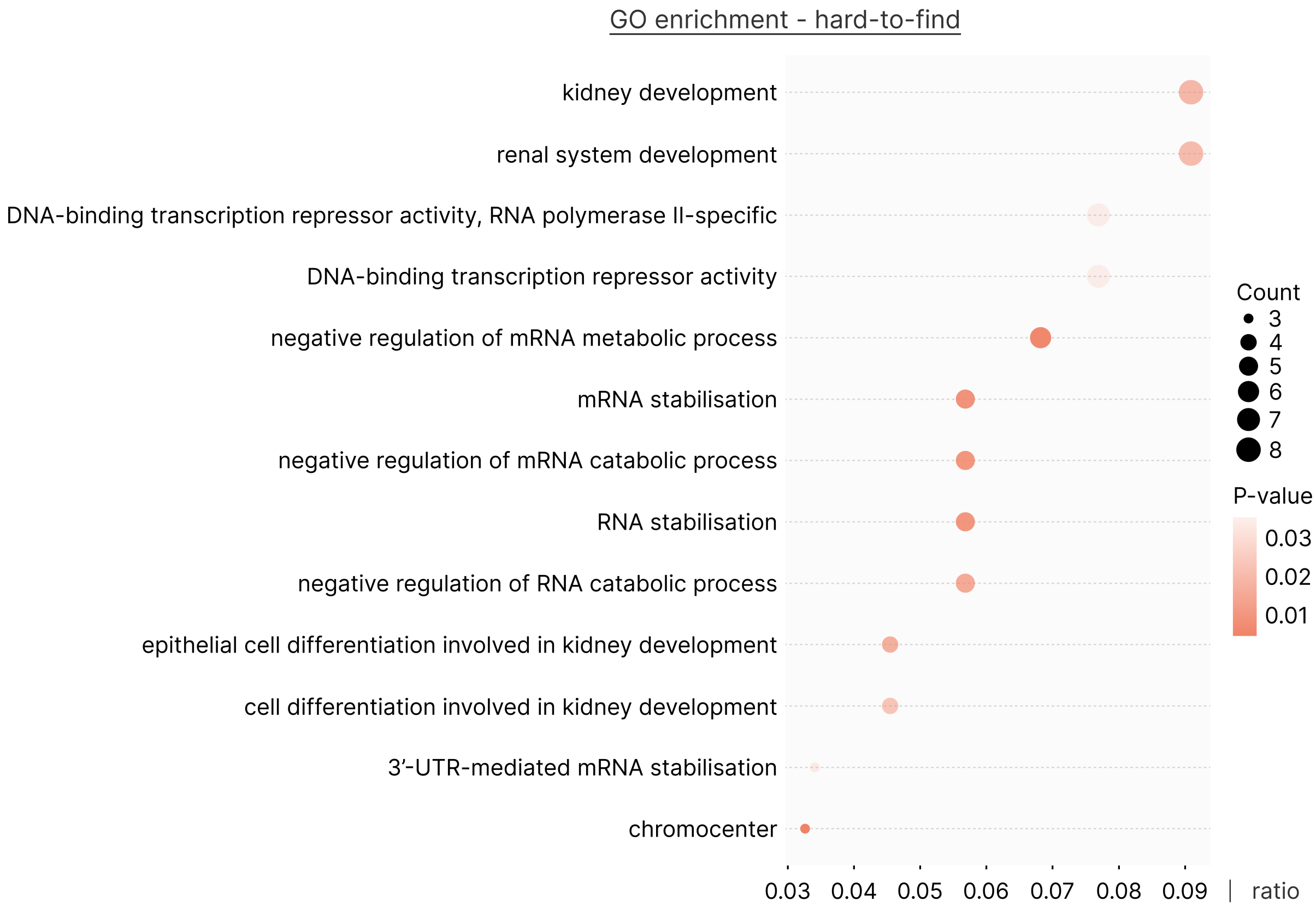

Fig. S3. GO term analysis for 'hard-to-find' ohnologs. This is a sub-set of the microsynteny-based ohnolog set. These ohnolog pairs fall below the usual threshold in a whole-genome similarity search.

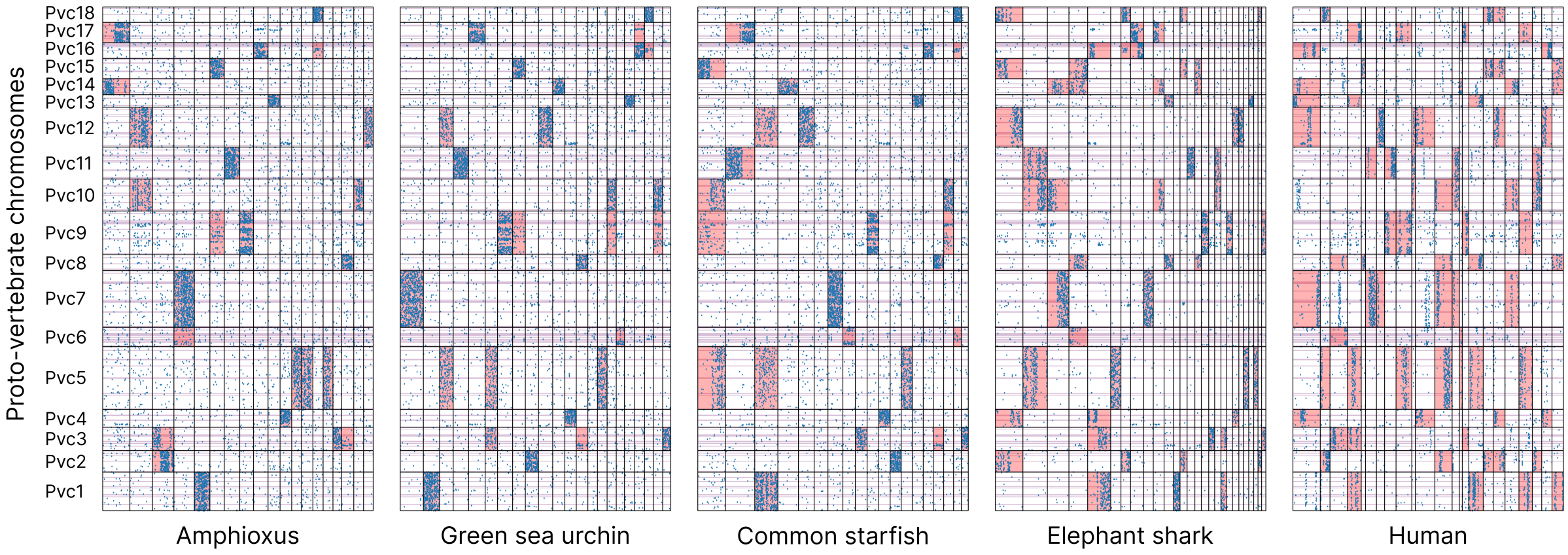

Fig. S4. Each of the reconstructed proto-vertebrate chromosomes exhibits a distinct ortholog distribution in invertebrate and gnathostome genomes. The reconstructed proto-vertebrate chromosomes represented by Japanese lamprey segments (y-axis), plotted against amphioxus, green sea urchin, common starfish, elephant shark, and human chromosomes (x-axis). Horizontal purple lines represent genomic segments in Japanese lamprey. Orthologous relationships are represented by blue dots. Chromosome relationships that are significantly enriched in orthologs are highlighted in red (see 'Methods').

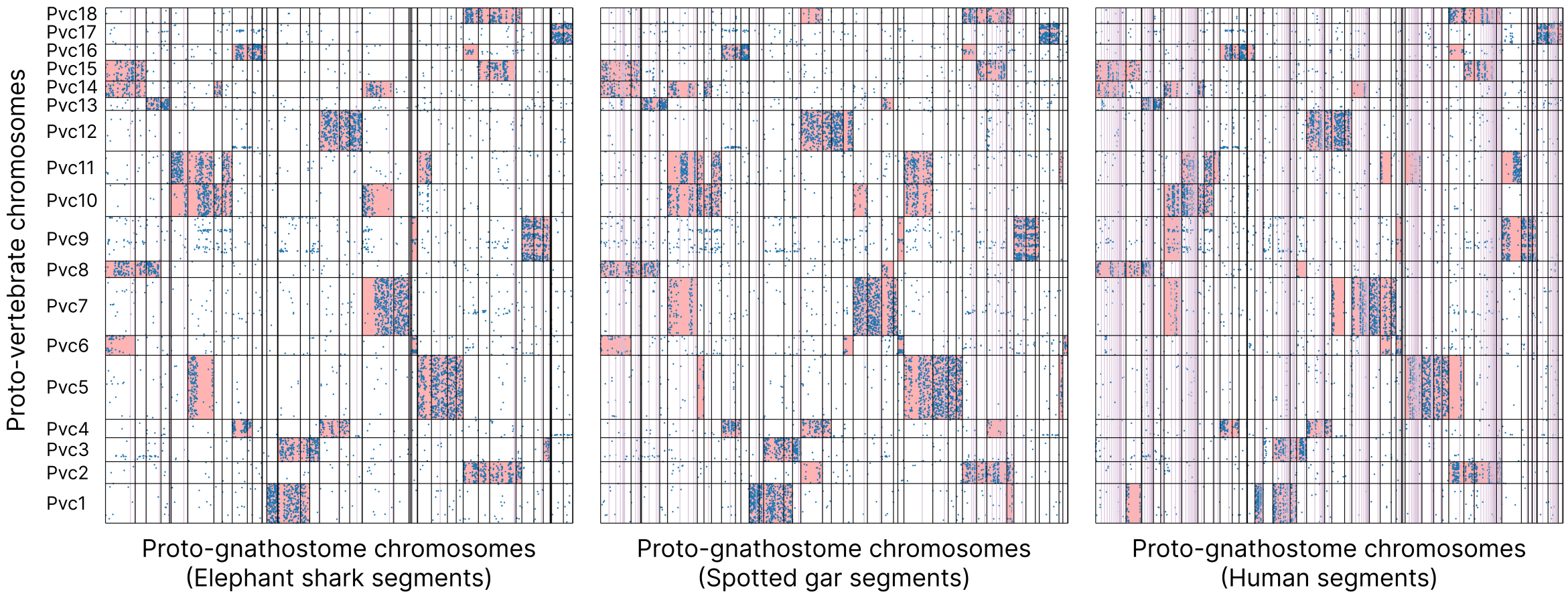

Fig. S5. Non-random distribution of orthologs between reconstructed pre-WGD proto-vertebrate chromosomes and post-WGD proto-gnathostome chromosomes. The reconstructed proto-vertebrate chromosomes represented by Japanese lamprey segments (y-axis), plotted against reconstructed proto-gnathostome chromosomes represented by elephant shark, spotted gar, and human segments (x-axis). Vertical purple lines represent genomic segments in gnathostome genomes. Orthologous relationships are represented by blue dots. Chromosome relationships that are significantly enriched in orthologs are highlighted in red (see 'Methods'). There is a one-to-four relationship between many pre-WGD and post-WGD chromosomes as expected given two consecutive rounds of WGD.

Table S1: Species used in our analysis. Each species that was used in our analysis is listed below. Extra information referenced throughout our methods is included. Of note, the Japanese lamprey proteome was downloaded from GitHub with duplicate protein isoforms already removed. Cyclostomes are assigned a multiplicity of 8 instead of 6 (26) for computational simplicity. Any non-WGD paralogs are removed in downstream filtering steps.

Species	Source	Group	Isoform method	Multiplicity
Roscoff worm	RefSeq	Invertebrate	Longest transcript	1
Purple sea urchin	RefSeq	Invertebrate	Longest transcript	1
Green sea urchin	RefSeq	Invertebrate	Longest transcript	1
Common starfish	RefSeq	Invertebrate	Longest transcript	1
Acorn worm	RefSeq	Invertebrate	Longest transcript	1
Ptychodera	OIST	Invertebrate	Longest transcript	1
Amphioxus	RefSeq	Invertebrate	Longest transcript	1
Sea squirt	RefSeq	Invertebrate	Longest transcript	1
Ciona	RefSeq	Invertebrate	Longest transcript	1
Sea lamprey	RefSeq	Cyclostome	Longest transcript	8
Japanese lamprey	GitHub	Cyclostome	Not applicable	8
Brook lamprey	RefSeq	Cyclostome	Longest transcript	8
Pacific lamprey	SIMRbase	Cyclostome	Longest transcript	8
Inshore hagfish	Ensembl	Cyclostome	Longest transcript	8
Brown hagfish	(5)	Cyclostome	Longest transcript	8
Elephant shark	RefSeq	Gnathostome	Longest transcript	4
Whale shark	RefSeq	Gnathostome	Longest transcript	4
Zebra shark	RefSeq	Gnathostome	Longest transcript	4
Little skate	RefSeq	Gnathostome	Longest transcript	4
Bichir	RefSeq	Gnathostome	Longest transcript	4
Bowfin	RefSeq	Gnathostome	Longest transcript	4
Spotted gar	Ensembl	Gnathostome	Longest transcript	4
Human	Ensembl	Gnathostome	Longest transcript	4
Mouse	Ensembl	Gnathostome	Longest transcript	4
Dog	Ensembl	Gnathostome	Longest transcript	4
Opossum	RefSeq	Gnathostome	Longest transcript	4
Chicken	(59)	Gnathostome	Longest transcript	4
Turkey	RefSeq	Gnathostome	Longest transcript	4
Zebra finch	RefSeq	Gnathostome	Longest transcript	4
Coelacanth	RefSeq	Gnathostome	Longest transcript	4
Green anole	RefSeq	Gnathostome	Longest transcript	4
Medaka	Ensembl	Gnathostome	Longest transcript	8
Pufferfish	Ensembl	Gnathostome	Longest transcript	8
Zebrafish	Ensembl	Gnathostome	Longest transcript	8
Stickleback	Ensembl	Gnathostome	Longest transcript	8
Sturgeon	GitHub	Gnathostome	Longest transcript	8

Table S2: Genome trios used for paralogy inference. For each group, the query genome and two references are shown. Only paralogs that are dated between the divergence of reference A and reference B are included. This should allow us to discard lineage-specific duplications. The number of top hits (N) that we keep for each trio is displayed. This reflects the number of duplicates we expect given the whole genome duplication history of the query species.

**Query species**	**Reference A**	**Reference B**	**N (multiplicity - 1)**
Sea lamprey	Amphioxus	Inshore hagfish	7
Japanese lamprey	Amphioxus	Inshore hagfish	7
Brook lamprey	Amphioxus	Inshore hagfish	7
Pacific lamprey	Amphioxus	Inshore hagfish	7
Inshore hagfish	Amphioxus	Sea lamprey	7
Brown hagfish	Amphioxus	Sea lamprey	7
Elephant shark	Amphioxus	Human	3
Whale shark	Amphioxus	Elephant shark	3
Zebra shark	Amphioxus	Elephant shark	3
Little skate	Amphioxus	Elephant shark	3
Bichir	Amphioxus	Elephant shark	3
Bowfin	Amphioxus	Elephant shark	3
Spotted gar	Amphioxus	Elephant shark	3
Human	Amphioxus	Elephant shark	3
Mouse	Amphioxus	Elephant shark	3
Dog	Amphioxus	Elephant shark	3
Opossum	Amphioxus	Elephant shark	3
Chicken	Amphioxus	Elephant shark	3
Turkey	Amphioxus	Elephant shark	3
Zebra finch	Amphioxus	Elephant shark	3
Coelacanth	Amphioxus	Elephant shark	3
Green anole	Amphioxus	Elephant shark	3
Medaka	Amphioxus	Elephant shark	7
Pufferfish	Amphioxus	Elephant shark	7
Zebrafish	Amphioxus	Elephant shark	7
Stickleback	Amphioxus	Elephant shark	7
Sturgeon	Amphioxus	Elephant shark	7

Table S3: Genome segmentation statistics. For each reconstruction, the genomes and various reconstruction parameters are shown. 'Max segments', 'alpha', and 'beta' are segmentation algorithm parameters. 'Window' defines the post-processing window size; A size of 5 includes the centre gene and two genes on either side. Cut-off values show the minimum number of breakpoint hits within a window that are required for a breakpoint to be kept. This value varies with the number of species in each group and the multiplicity of those species. The pre- and post-processed breakpoint and segment numbers, respectively, are given.

Proto-vertebrate reconstruction

**Species**	**Max segments**	**Alpha**	**Beta**	**Window**	**Cut-off 1**	**Cut-off 2**	**Breakpoints**	**Segments**
Amphioxus	20	1.0	1.0	5	2	5	171	52
Sea lamprey	20	1.0	1.0	5	2	5	513	211
Japanese lamprey	20	1.0	1.0	5	2	5	197	158
Brook lamprey	20	1.0	1.0	5	2	5	484	140
Pacific lamprey	20	1.0	1.0	5	2	5	229	143
Inshore hagfish	20	1.0	1.0	5	2	5	1229	330
Brown hagfish	20	1.0	1.0	5	2	5	1123	323

Table S4: Genome segmentation statistics. For each reconstruction, the genomes and various reconstruction parameters are shown. 'Max segments', 'alpha', and 'beta' are segmentation algorithm parameters. 'Window' defines the post-processing window size; A size of 5 includes the centre gene and two genes on either side. Cut-off values show the minimum number of breakpoint hits within a window that are required for a breakpoint to be kept. This value varies with the number of species in each group and the multiplicity of those species. The pre- and post-processed breakpoint and segment numbers, respectively, are given.

Proto-gnathostome reconstruction

**Species**	**Max segments**	**Alpha**	**Beta**	**Window**	**Cut-off 1**	**Cut-off 2**	**Breakpoints**	**Segments**
Amphioxus	20	1.0	1.0	5	5	8	311	43
Elephant shark	20	1.0	1.0	5	5	8	2225	71
Whale shark	20	1.0	1.0	5	5	8	2349	87
Zebra shark	20	1.0	1.0	5	5	8	3172	102
Little skate	20	1.0	1.0	5	5	8	2878	91
Bichir	20	1.0	1.0	5	5	8	3812	138
Bowfin	20	1.0	1.0	5	5	8	3252	82
Spotted gar	20	1.0	1.0	5	5	8	3138	88
Human	20	1.0	1.0	5	5	8	4055	202
Mouse	20	1.0	1.0	5	5	8	4879	226
Dog	20	1.0	1.0	5	5	8	4085	203
Opossum	20	1.0	1.0	5	5	8	3179	120
Chicken	20	1.0	1.0	5	5	8	2378	69
Turkey	20	1.0	1.0	5	5	8	2393	101
Zebra finch	20	1.0	1.0	5	5	8	2957	89
Coelacanth	20	1.0	1.0	5	5	8	2854	76

Table S5: Significance of reconstruction for the ancestral vertebrate. The significance of reconstructions containing K pre-WGD chromosomes. The log transformed probabilities calculated for jawless vertebrate segments are shown (see Equation 3). The probabilities have been rounded to two decimal places. The most significant number of ancestral vertebrate chromosomes, $K = 18$, is highlighted in bold.

K	Sea lamprey	Japanese lamprey	Brook lamprey	Pacific lamprey	Inshore hagfish	Brown hagfish
10	-6698.84	-7441.34	-7908.30	-3464.81	-4463.91	-2542.19
11	-6729.98	-7455.67	-8133.07	-3591.68	-4569.70	-2554.00
12	-7302.51	-7837.42	-8497.58	-3747.50	-4954.20	-2734.46
13	-7796.85	-8361.77	-9133.36	-3827.13	-5112.21	-2867.33
14	-8002.72	-8542.07	-9497.04	-3867.45	-5060.12	-2984.10
15	-8288.80	-8674.28	-9867.82	-3918.83	-5120.36	-3014.94
16	-8379.40	-8731.17	-9992.46	-3975.12	-5189.69	-3107.39
17	-8573.16	-8913.54	-10444.38	-4128.99	-5527.92	-3199.76
**18**	**-8623.79**	**-8913.90**	**-10494.70**	**-4171.81**	**-5677.16**	**-3287.47**
19	-8016.09	-8740.03	-9738.04	-3976.27	-5436.90	-3086.69
20	-6963.70	-7756.50	-8717.46	-3257.01	-4990.93	-3041.78
21	-6780.26	-7739.77	-8760.24	-3121.59	-4855.90	-2970.74
22	-6512.27	-7437.07	-8247.55	-3033.79	-4742.99	-3009.08
23	-6279.95	-7040.05	-7959.98	-2780.71	-4678.84	-2942.75
24	-6014.81	-6718.67	-7729.79	-2602.12	-4373.62	-2939.35
25	-5678.11	-6131.64	-6970.40	-2325.15	-4206.27	-2784.36
26	-5549.45	-5852.58	-6913.66	-2178.12	-4222.42	-2740.58
27	-5298.50	-5743.37	-6925.00	-2039.95	-3803.88	-2542.77
28	-5403.34	-5760.03	-6931.69	-2161.93	-4134.37	-2664.54
29	-5093.02	-5669.79	-6950.68	-2044.40	-3800.39	-2442.02
30	-5205.38	-5587.48	-6888.95	-1997.40	-3825.71	-2468.77

Table S6: Significance of reconstruction for the initial step in the ancestral gnathostome reconstruction. The significance of reconstructions containing K pre-WGD chromosomes. The log transformed probabilities calculated for jawed vertebrate segments are shown (see Equation 3). The probabilities have been rounded to the nearest integer. The most significant number of ancestral vertebrate chromosomes for each species is highlighted in bold.

**K**	*Elephant shark*	*Whale shark*	*Zebra shark*	*Little skate*	*Bichir*	*Bowfin*	*Spotted gar*	*Human*	*Mouse*	*Dog*	*Opossum*	*Chicken*	*Turkey*	*Zebra finch*	*Coelacanth*
5	-7727	-9648	-10391	-8202	-9822	-10282	-8231	-8205	-7945	-5898	-7044	-8582	-6990	-8030	-11547
6	-8207	-10294	-11599	-8921	-10791	-11272	-9048	-8664	-8757	-6523	-7841	-9406	-7701	-8318	-12863
7	-8193	-10773	-12607	-9536	-10831	-10812	-9059	-10062	-10274	-7564	-8791	-9953	-8180	-9405	-13340
8	-8625	-11094	-12830	-9900	-11535	-11748	-9722	-10581	-10684	-7843	-9168	-10343	-8639	-9743	-13845
9	-8920	-11495	-13305	-10205	-11721	-11891	-9977	-10993	-11053	-8027	-9310	-10683	-8943	-9971	-14379
10	**-9047**	**-11866**	**-13562**	**-10257**	-12074	-12449	**-10111**	**-11103**	**-11668**	-8029	**-9458**	**-10978**	**-9050**	**-10134**	**-14810**
11	-8577	-11379	-13180	-9930	-12552	-12419	-9860	-11028	-11621	**-8095**	-9279	-10415	-8713	-9766	-14548
12	-8301	-11031	-12458	-9484	**-12664**	**-12781**	-9689	-10679	-10913	-7793	-8862	-9891	-8408	-9172	-14058
13	-7155	-9975	-11396	-8255	-11179	-11371	-8474	-10094	-10363	-7143	-8073	-9346	-7278	-8225	-12727
14	-6774	-9495	-10910	-7785	-10678	-10872	-8112	-10232	-10393	-7117	-7928	-9019	-6993	-7813	-12110
15	-5968	-9278	-10496	-7397	-10310	-9916	-7569	-9788	-10161	-7035	-7682	-8061	-6604	-7558	-11622
16	-5757	-9270	-10626	-7386	-10595	-9913	-7554	-10192	-10496	-7449	-7410	-8183	-6182	-7558	-11675
17	-5126	-9099	-10564	-7006	-10532	-9780	-7577	-9999	-10399	-7353	-6861	-7857	-5474	-7272	-10999
18	-5168	-8338	-9981	-6505	-10264	-9243	-7308	-9666	-10024	-7105	-7241	-7926	-5393	-7036	-10420
19	-4571	-7381	-9002	-5418	-9474	-8783	-6505	-8634	-8787	-6278	-6327	-6956	-4707	-6125	-9099
20	-4121	-6930	-8580	-5100	-8962	-8405	-6004	-7932	-8203	-5697	-5454	-6520	-4195	-5719	-8618
21	-3366	-6414	-7778	-4823	-9316	-7811	-5665	-7610	-8445	-5758	-6050	-6349	-3840	-5507	-8444
22	-3320	-6002	-7381	-4282	-8623	-7454	-5350	-6999	-8046	-5194	-5588	-5858	-3482	-5030	-7775
23	-2880	-5718	-7185	-4110	-8244	-7108	-4885	-6573	-7443	-4757	-4883	-5478	-3269	-4687	-7412
24	-2880	-5747	-7147	-4110	-8127	-7149	-4885	-6573	-7507	-4836	-4883	-5478	-3264	-4687	-7412
25	-2880	-5747	-7205	-4110	-8127	-7149	-4919	-6445	-7388	-4735	-4883	-5478	-3264	-4661	-7412
